# Successful management of recalcitrant cutaneous warts with low-dose acitretin monotherapy in a patient with idiopathic CD4^+^ lymphocytopenia

**DOI:** 10.1093/skinhd/vzaf025

**Published:** 2025-06-03

**Authors:** Moe Latt, Alex Langrish, Alison M Layton, Charles J N Lacey

**Affiliations:** Department of Dermatology, Harrogate and District Hospital NHS Foundation Trust, Harrogate, UK; Department of Genitourinary Medicine, York and Scarborough Teaching Hospitals NHS Foundation Trust, York, UK; Department of Dermatology, Harrogate and District Hospital NHS Foundation Trust, Harrogate, UK; Skin Research Centre, University of York, York, UK; York Biomedical Research Institute, Hull York Medical School, University of York, York, UK; York Biomedical Research Institute, Hull York Medical School, University of York, York, UK

## Abstract

A 50-year-old man with a background of idiopathic CD4^+^ lymphocytopenia (ICL) was successfully treated with acitretin monotherapy for recalcitrant viral warts on the hands and right cheek. Treatment duration was 35 months, with sustained effects at 15 months post-acitretin therapy. Side-effects were mild and included mild cheilitis and dryness of nasal mucosa. CD4^+^ lymphocytes play an important role in enabling host responses to human papillomavirus (HPV) and, consequently, lower CD4 counts correspond to a higher risk of chronic HPV infection, including viral warts. HPV represents the most common opportunistic infection in patients with ICL. Acitretin is an oral retinoid with affinity for retinoic acid and retinoid X receptors, and works through downregulation of signal transducer and activator of transcription (STAT)1- and STAT3-dependent signalling, with resultant increases in keratinocyte differentiation. As HPV evades the Janus kinase/STAT pathway to promote keratinocyte proliferation, acitretin may work through reversal of this mechanism. This case demonstrates effectiveness of acitretin in treating recalcitrant viral warts, specifically in a patient with ICL.

## Teaser

A case demonstrating sustained effectiveness of acitretin monotherapy in treating recalcitrant viral warts in a patient with idiopathic CD4 lymphocytopenia.

**What is already known about this topic?**

- Human papillomavirus (HPV) infection is common in immunocompromised patients.
- HPV warts can be unresponsive to many treatment modalities.

**What does this study add?**

- Acitretin is a well-tolerated and effective treatment option for recalcitrant viral warts in the context of immunocompromise.

## Case report

A 50-year-old man was referred to dermatology from genitourinary medicine with recalcitrant, hyperkeratotic viral warts on the right cheek and dorsum of the hands, present for >10 years’ duration. The initial wart appeared on the right knuckle 23 years ago and enlarged gradually, with subsequent warts appearing over time, carrying a predilection for the face and hands.

His medical history was notable for a diagnosis of idio­pathic CD4^+^ lymphocytopenia (ICL), formalized 9 years previously. There was no family history of ICL or viral warts, and no other medical morbidity. Historic treatments included hyfrecation of the wart on the right cheek and multiple cycles of liquid nitrogen cryotherapy. Topical imiquimod 5% cream and salicylic acid 40% had been used on the digital warts, with limited effect. He had also taken oral isotretinoin for 2 years, resulting in some improvement of the facial warts, but which had not led to complete ­resolution.

**Figure 1:**
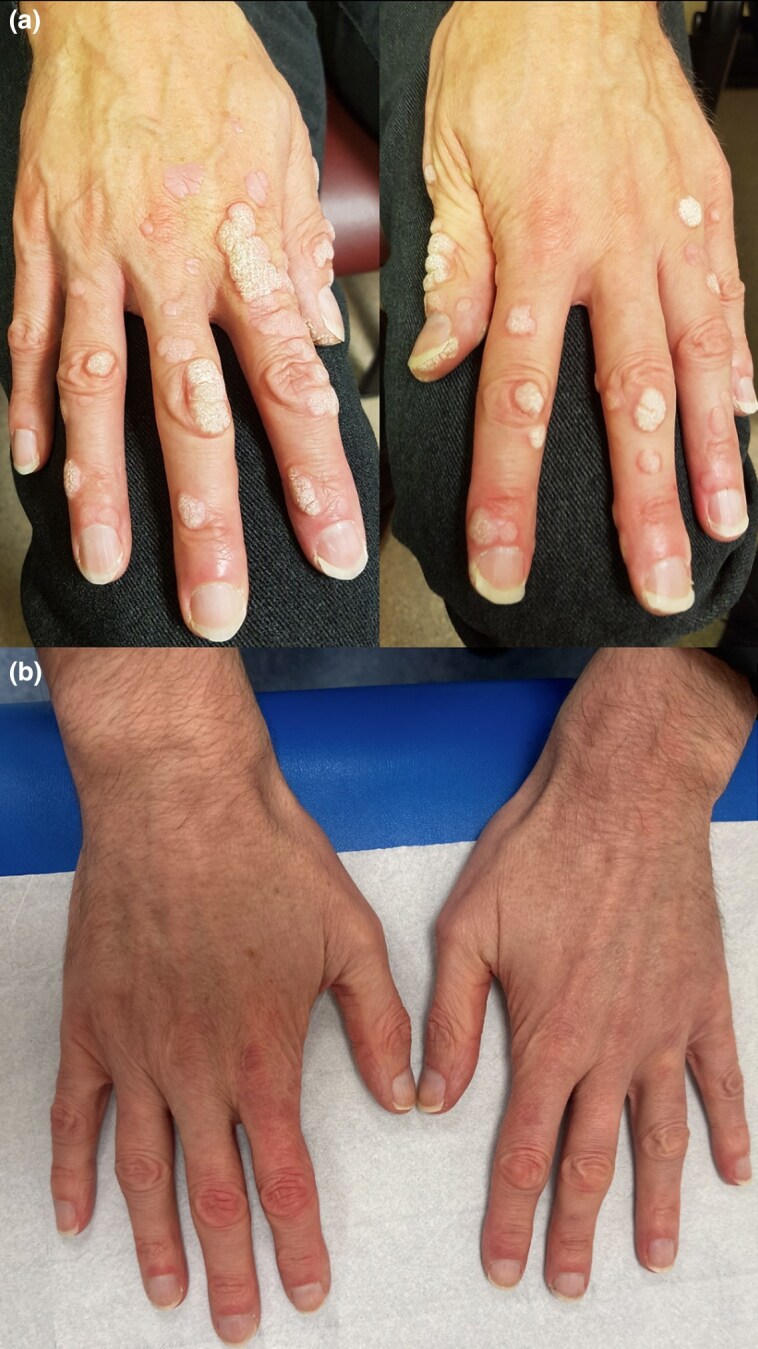
(a) Human papillomavirus warts on the dorsum of the hands at baseline and (b) 3 months after stopping acitretin therapy.

**Figure 2:**
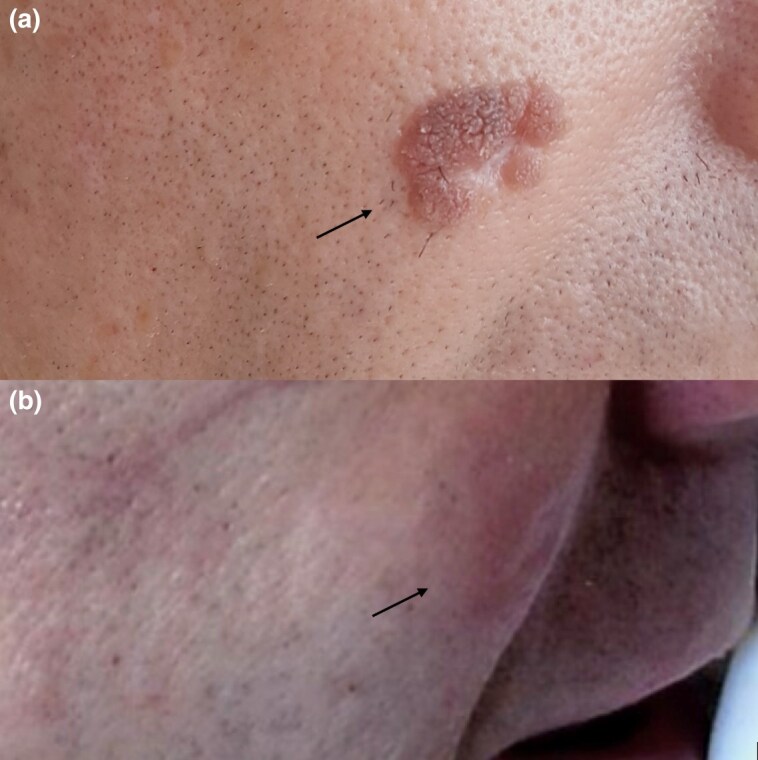
(a) Human papillomavirus wart on the right cheek at baseline and (b) 3 months after cessation of acitretin therapy.

Physical examination revealed numerous, coalescing flat warts on the dorsal aspect of the hands and all 10 digits, diagnosed clinically. The wart on the right mid-cheek was violet-red in hue (Figures 1, 2).

Baseline blood work, including full blood count, urea and electrolytes, liver function, lipids and haemoglobin A1c were within normal limits. Total lymphocyte count was 775 × 10^6^ cells L^–1^ [CD3 45%, CD4 21% (total CD4 152 × 10^6^ cells L^–1^), CD8 22%, CD19 30% and natural killer 25%]. HIV antigen/antibody was negative on multiple occasions.

Oral acitretin was the treatment selected due to the widespread nature and location of the warts, accounting for prior failed treatments. There have also been previous favourable outcomes with acitretin use in treating multiple recalcitrant viral warts,^1^ and success specifically in a patient with ICL.^2^

Acitretin was started initially at 10 mg once daily (0.13 mg kg^–1^) and titrated upwards over a 6-month period to 25 mg once daily (0.3 mg kg^–1^). A low-dose approach (defined as ≤25 mg daily) was favoured, as the patient had previously developed clinically significant xerosis on oral isotretinoin. Lower doses of acitretin have been suggested to carry fewer side-effects, such as xerosis and cheilitis.^3^ He was maintained on 25 mg once daily for 23 months until initial resolution, then titrated down to 25 mg on alternating days for a further 6 months (0.16 mg kg^–1^), prior to stopping. He was followed up at 3 and 15 months post-treatment cessation.

The patient was informed of the risks of acitretin use prior to initiation, and regular monitoring of serum lipid profile and liver function was implemented. The total duration of oral acitretin treatment was 35 months, with only minor adverse effects (mild cheilitis and dryness of nasal mucosa) and no derangement in blood tests during this period. The patient had no recurrence of disease at 3 (Figures 1, 2) and 15 months post-treatment.

## Discussion

ICL is a rare condition defined as a CD4^+^ T-cell count of <300 cells mm^–3^ or <20% of total lymphocytes measured at least 6 weeks apart in the absence of disease or treatment associated with immunodeficiency.^4^ This patient was initially diagnosed with ICL by the infectious diseases team, when he presented with presumptive *Pneumocystis jirovecii* pneumonia. Since then, his CD4 count had consistently measured <200 × 10^6^ cells L^–1^. Aside from prophylactic oral co-trimoxazole (480 mg once daily), he had not received any other treatments for ICL.

Human papillomaviruses (HPV) consist of 5 genera and >170 types within the family Papillomaviridae. They are small, nonenveloped, double-stranded DNA viruses that enter the skin through minor epithelial trauma and infect the basal epithelium. Infection with HPV can result in a range of benign and cancerous lesions, including warts, on skin and epithelial surfaces. HPV uses the epithelial cell cycle to replicate and propagates through natural shedding of the stratum corneum.^5^ Chronic infection with certain HPV genotypes is a known a etiological factor for oncogenesis, although this is also dependent on genetic, immunological, mutagenic and environmental factors.^6^

CD4^+^ lymphocytes are among the major immune factors implicated in the response to chronic HPV infection, although the mechanisms remain incompletely understood.^5^ CD4^+^ lymphocytes are important in enabling responses to HPV and, consequently, reduced numbers of CD4^+^ lymphocytes are associated with a higher risk of persistent HPV infection.^5^

The largest cohort study of patients with ICL recently showed that HPV was the most common opportunistic infection, and the risk of opportunistic infection within ICL was directly related to CD4 count.^6^ Similarly, a study using metagenomic sequencing demonstrated that patients with ICL carry higher readings of HPV in the skin when compared with healthy controls, suggesting that the condition has a differential effect on the skin microbiome.^7^

Acitretin is a second-generation retinoid, administered orally. Acitretin is isomerized to isoacitretin and exerts its effects through binding to retinoid acid and retinoid X receptors to alter gene transcription, downregulating signal transducer and activator (STAT)1- and STAT3-dependent signalling.^8,9^ The resultant effect is decreased keratinocyte proliferation and increased cellular differentiation; consequently, acitretin is of use in treating disorders of keratinization, such as viral warts.^8,9^ HPV is known to manipulate the Janus kinase/STAT pathway to evade the immune system and promote cellular proliferation, and it seems likely that acitretin reverses both these effects.^10^ Increasing concentrations of retinoids have also been shown to have an inverse relationship with HPV–DNA content, advocating for an additional effect of this medication on HPV replication.^11^

Treatment options for HPV warts are numerous and include combination or monotherapy of topical treatments, physical destruction techniques, immunotherapy or retinoids.

Topical therapies include salicylic acid, cantharidin, podophyllin, 5-fluorouracil and topical imiquimod, which cause irritation, ablation and trigger a primary immune response to enable elimination of HPV-infected cells.^12^

Photodynamic therapy, including conventional or daylight with topical aminolaevulinic acid, can be used. Photodynamic therapy works through targeting excess quantities of protoporphyrin IX produced in cells infected with HPV to cause cellular damage and apoptosis.^13^

Cryotherapy, CO_2_ laser and electrocautery may be used to physically destroy affected tissue. Surgical excision is also an option for select lesions.^12^

Immunotherapy, through topical application of diphencyprone, intralesional injection of Candida, Trichophyton or mumps antigen, bleomycin or vaccinations (e.g. HPV; measles, mumps and rubella; Mycobacterium w and tuberculin purified protein derivative, have also been described.^14^

Given the large number of potential treatment options for HPV warts, careful selection and treatment escalation is required, with consideration of the risk–benefit ratio in the individual patient.

This case highlights the clinical benefit of acitretin use in recalcitrant HPV associated with immunodeficiency, specifically in the context of a patient with ICL and using low-dose treatment. Such patients are at higher risk of chronic HPV infection and subsequent malignancy and respond suboptimally to many other treatment options. This patient demonstrated no significant side-effects on 35 months of acitretin and remained symptom-free at the 15-month ­follow-up. Acitretin should be considered for treatment of refractory HPV warts and is a valid option in the immunocompromised.

## Data Availability

The data are available in the article.
